# Beyond fluid intelligence and personality traits in social support: the role of ability based emotional intelligence

**DOI:** 10.3389/fpsyg.2015.00395

**Published:** 2015-04-08

**Authors:** Annamaria Di Fabio

**Affiliations:** Department of Education and Psychology, University of FlorenceFlorence, Italy

**Keywords:** fluid intelligence, personality traits, ability based emotional intelligence, social support, prevention

## Abstract

Social support represents an important individual resource that has been associated with multiple indices of adaptive functioning and resiliency. Existing research has also identified an association between emotional intelligence (EI) and social support. The present study builds on prior research by investigating the contributions of ability based EI to social support, beyond the effects of fluid intelligence and personality traits. The Advanced Progressive Matrices, the Big Five Questionnaire, the Mayer Salovey Caruso EI test (MSCEIT), and the Multidimensional Scale of Perceived Social Support were administered to 149 Italian high school students. The results showed that ability based EI added significant incremental variance in explaining perceived social support, beyond the variance due to fluid intelligence and personality traits. The results underline the role of ability based EI in relation to perceived social support. Since ability based EI can be increased through specific training, the results of the present study highlight new possibilities for research and intervention in a preventive framework.

## Introduction

Social support represents an important human resource that can be understood from multiple theoretical perspectives (Di Fabio and Kenny, 2012b; Blustein and Di Fabio, 2013). Relational theory (Blustein, 2001), for example, highlights the value of social support, noting the natural human aspiration for personal connection, and relationships (Bowlby, 1982; Josselson, 1992; Blustein, 2001, 2006). The aspiration for connection is evident across the life span through friendships, romantic relationships, familial connections, and work relationships (Blustein, 2006). Research has documented the importance of relational experiences and interpersonal relationships both for career development and work success (Blustein et al., 1995; Schultheiss et al., 2001; Kenny et al., 2003; Blustein, 2006; Richardson, 2012). According to the inclusive psychology of working (Blustein, 2011), work is an inherently relational act such that each decision, experience, and interaction with the world of work is understood, influenced and shaped by relationships. Accordingly, career development interventions now highlight the importance of relationships in designing a career or Career project (Savickas, 2011) and as integral to the Life project (Guichard, 2013).

Social support also serves important preventive and protective functions in fostering positive development and buffering stress (Hage et al., 2007; Kenny and Di Fabio, 2009; Kenny and Hage, 2009; Hage and Romano, 2013; Kenny et al., 2014; Di Fabio and Kenny, 2015). Research has found social support to be related to quality of life (Petito and Cummins, 2000; Helgeson, 2003) and well-being (Rigby, 2000; Ben-Ari and Gil, 2004). Evidence suggests, furthermore, that positive relationships can help to buffer the negative effects of psychosocial stress and job loss (Aquino et al., 1996; Blustein et al., 1997; Greenhaus and Parasuraman, 1999; Blustein, 2006). Relationships thus offer valuable resources for responding to the challenges of the 21st century and in helping people to build their work lives (Hage et al., 2007; Richardson, 2012; Kenny et al., 2014; Di Fabio and Kenny, 2015). The positive youth development (Lerner, 2001; Kenny, 2007, 2014) and developmental–contextual frameworks (Walsh et al., 2002) also emphasize the important role of the social context at the family, school, and community levels in moderating risks and fostering academic success and productive societal participation (Dryfoos, 1994; Paavola et al., 1995; Airasian and Walsh, 1997; Schorr, 1997; Ferry et al., 2000; Kenny et al., 2014; Di Fabio and Kenny, 2015). Building supportive relationships is thus important for prevention efforts in early life and across the life course (Hage et al., 2007; Kenny and Hage, 2009; Blustein, 2011).

Positive Lifelong Self and Relational Management (PLS&RM, Di Fabio, 2014a; Di Fabio et al., in press) is a new theoretical integration that emanates from Positive Psychology (Seligman and Csikszentmihalyi, 2000; Seligman, 2002), brings into consideration the dialectic of self in relationship, and is aligned with developmental-contextualism (Lerner, 2002; Kenny, 2007), career construction (Savickas, 2005), self-construction and life-construction (Guichard, 2005, 2013), and the relational theory of working (Blustein, 2011). PLS&RM refers to the promotion of effective and lifelong self and relational management across numerous personal and professional transitions and complex challenges of 21st century life. PLS&RM is comprised of three constructs: Positive Lifelong Life Management, Positive Lifelong Self Management, and Positive Lifelong Relational Management. The first construct is operationalized by the Pemberton Happiness Index (PHI, Hervás and Vázquez, 2013) and the Authenticity Scale (AS, Wood et al., 2008); the second construct by the Intrapreneurial Self-Capital Scale (ISC, Di Fabio, 2014b), the Career Adapt-Abilities Inventory (Savickas and Porfeli, 2012), and the Life Project Reflexivity Scale (Di Fabio, in press b); the third construct by the Trait Social-Emotional Intelligence Questionnaire (TEIQue, Petrides and Furnham, 2004; the Bar-On EQ-I, Bar-On, 1997), the Multidimensional Scale of Perceived Social Support (MSPSS, Zimet et al., 1988), and the Positive Relational Management Scale (Di Fabio, in press c), encompassing respect for others, respect by others for oneself, respect for oneself; caring for others, caring by others for oneself, caring for oneself; and connection with family, connection with friends, connection with significant others.

Following a preventive orientation, PLS&RM (Di Fabio, 2014a; Di Fabio et al., in press) focuses on building strengths at the person (Di Fabio and Blustein, 2010; Di Fabio and Kenny, 2011, 2012a; Di Fabio and Palazzeschi, 2012; Di Fabio et al., 2012, 2013; Kenny et al., 2014; Di Fabio, in press a) and relational levels (Blustein, 2006, 2011; Di Fabio and Kenny, 2012b). Given prior research on the value of social support for prevention and positive development, social support is of clear relevance to the PLS&RM model as a strength for promoting positive lifelong management and wellness.

Social support has been widely researched over the years, with attention to its relationship with personality traits. Early research established positive relationships with extraversion and negative relationships with neuroticism (Sarason et al., 1983, 1986). More recent research has documented positive relationships between perceived social support and the personality traits of agreeableness and conscientiousness (Di Fabio and Kenny, 2012b). Recent research has also explored the relationship of perceived social support with emotional intelligence (EI; Austin et al., 2005; Montes-Berges and Augusto, 2007; Di Fabio and Kenny, 2012b). While EI is considered a variable that can be increased through specific training with potential for prevention (Di Fabio and Kenny, 2011), personality traits are considered generally stable and less amenable to modification through intervention (Costa and McCrae, 1992). Given evidence that EI is associated with perceived social support (Di Fabio and Kenny, 2011, 2012b), EI has promise for promoting personal well-being and PLS&RM.

The term EI was introduced by Salovey and Mayer (1990) to describe the individual’s ability to monitor his or her own feelings and those of others, discriminating among the various types of emotions and utilizing this information to guide thoughts and actions. Salovey and Mayer (1990) affirm that EI is composed of three categories of adaptive abilities: Appraisal and expression of emotions, Regulation of emotions, Utilization of emotions in solving problems. Subsequently Mayer and Salovey (1997) expanded their definition to include the ability to perceive emotions, to compare emotions and feelings connected to them, to understand information derived from emotions, and to manage these emotions. Saklofske et al. (2003) and Stough et al. (2009) distinguish two principal EI models: ability based models refer to EI abilities (Mayer et al., 2000) and trait EI models refer to self-reported EI (Bar-On, 1997) and EI self-efficacy (Petrides and Furnham, 2000, 2001).

Recent research on EI (Di Fabio and Palazzeschi, 2008a,b, 2009; Di Fabio and Blustein, 2010; Di Fabio and Kenny, 2012a; Di Fabio et al., 2012, 2013; Di Fabio and Saklofske, 2014a,b) has examined the incremental contribution of EI to varied indices of adaptive functioning beyond the contribution of personality traits. Prior research has established relationships between trait EI and perceived social support (Austin et al., 2005; Montes-Berges and Augusto, 2007; Di Fabio and Kenny, 2012b) and between ability based EI and perceived social support (Di Fabio and Kenny, 2012b).

### Aim and Hypotheses

Following from the theoretical frameworks and prior research delinated above, social support represents an important individual resource associated with multiple indices of adaptive functioning (Blustein et al., 1997; Petito and Cummins, 2000; Rigby, 2000; Helgeson, 2003; Ben-Ari and Gil, 2004; Blustein and Di Fabio, 2013). Social support also represents an important preventive and protective factor in fostering positive development (Hage et al., 2007; Kenny and Di Fabio, 2009; Kenny and Hage, 2009; Hage and Romano, 2013; Kenny et al., 2014; Di Fabio and Kenny, 2015). Existing research has also identified a specific association between EI and social support (Austin et al., 2005; Montes-Berges and Augusto, 2007; Di Fabio and Kenny, 2012b). The present study builds on prior research by investigating the contribution of ability based EI to social support, beyond the effects of fluid intelligence and personality traits in Italian high school students attending the last year. The decision to examine these aspects in a sample of students attending the last year of an Italian high school was determined by this critical stage in the life of the participants examined, since they had to cope with important choices and transitions at the end of high schoolregarding their development and career. The following two hypotheses were formulated.

H1: Personality traits will explain a significant percentage of incremental variance over variance explained by fluid intelligence in relation to perceived social support (Sarason et al., 1983, 1986; Di Fabio and Kenny, 2012b).

H2: Ability based EI will explain a significant percentage of incremental variance over the variance due to fluid intelligence and personality traits in relation to perceived social support (Di Fabio and Kenny, 2012b).

## Materials and Methods

### Participants

One hundred and forty-nine students attending the last year of high school in the Tuscan school system participated in the study. All final-year high school students in the school system were invited to participate. With regard to gender, 58 (38.41%) of the participants were boys and 91 (60.26%) were girls. The participants ranged in age from 18 to 20 years (*M* = 19.48, SD = 0.56).

### Measures

#### Advanced Progressive Matrices (APM)

The Italian version by Di Fabio and Clarotti (2007) of the Advanced Progressive Matrices (APM) test by Raven (1962) was used to evaluate fluid intelligence. The test is composed of two series of items, with12 items in Series I and 36 items in Series II. Participants select one response for each item among eight possible alternatives. Cronbach’s alpha was 0.91.

#### Big Five Questionnaire (BFQ)

The Big Five Questionnaire (BFQ, Caprara et al., 1993) was used to evaluate personality traits. The questionnaire is composed of 132 items with response options on a 5-point Likert scale format ranging from 1 = *Absolutely false* to 5 = *Absolutely true*. The questionnaire measures five personality dimensions. In the Italian sample, the Cronbach’s alpha coefficients were:0.81 for Extraversion, 0.73 for Agreeableness, 0.81 for Conscientiousness, 0.90 for Emotional Stability, and 0.75 for Openness.

#### Mayer Salovey Caruso Emotional Intelligence Test (MSCEIT)

The Italian version by D’Amico and Curci (2010) of the Mayer Salovey Caruso EI Test (MSCEIT, Mayer et al., 2002) was used to evaluate ability based EI. The measure has 141 items and provides a total score and four branch scores: Perceiving Emotions (PE), Facilitating Thought (FT), Understanding Emotions (UE), Managing Emotions (ME). For the Italian version, split half reliabilities were: 0.90 for PE, 0.77 for FT, 0.75 for UE, 0.72 for ME (D’Amico and Curci, 2010).

#### Multidimensional Scale of Perceived Social Support (MSPSS)

The Italian version by Di Fabio and Busoni (2008) of the Multidimensional Scale of Perceived Social Support (MSPSS, Zimet et al., 1988) was used to evaluate perceived social support. The scale is composed of 12 items with response options on a 7-point Likert-type scale, ranging from 1 (*absolutely false*) to 7 (*absolutely true*). The instrument measures support from family (Example of item: “My family works very hard to help me”), friends (Example of item: “I can speak about my problems with my friends”), and significant others (Example of item: “When I need someone, there is always a special person who stands by me”). For the Italian version, the Cronbach’s alpha coefficient was 0.90.

### Procedure and Data Analysis

The measures were administered collectively in the classroom by trained staff. The administration order was counterbalanced to check for potential presentation order effects.

The measures were administered at a time agreed upon with the school and with due adherence to the requirements of privacy and informed consent requested by the Italian law (Law Decree DL-196/2003). Regarding the ethical standards for research, the study referred to the last version of the Declaration of Helsinki (Fortaleza, 2013).

Descriptive statistics, Pearson’s *r* correlation and hierarchical regressions were calculated for the data.

## Results

Means, SDs and correlations between APM, BFQ, MSCEIT, and MSPSS are reported in **Table 1**.

**Table 1:** Means, SDs, and correlations relative to APM, BFQ, MSCEIT, MSPSS.

		*M*	SD	1	2	3	4	5	6	7	8
1	APM	34.94	6.57	–							
2	BFQ Extraversion	76.02	10.63	0.02	–						
3	BFQ Agreeableness	76.26	9.21	0.09	0.34**	–					
4	BFQ Consciousness	78.96	9.63	0.09	0.28**	0.23**	–				
5	BFQ Emotional Stability	61.69	13.81	0.10	0.39**	0.32**	0.31**	–			
6	BFQ Openness	79.25	9.36	0.13	0.42**	0.39**	0.37**	0.40**	–		
7	MSCEIT total score	42.74	7.05	0.19	0.09	0.07	0.08	0.10	0.11	–	
8	MSPSS total score	42.23	8.35	0.05	0.35**	0.24**	0.21**	0.32**	0.29**	0.52**	–

*N* = 149, **p < 0.01.

With the MSPSS as criterion variable, a hierarchical regression was conducted with fluid intelligence at the first step, personality traits at the second step, and ability based EI at the third step (see **Table 2**).

**Table 2:** Hierarchical regression.

	MSPSS total score
	**β**
*Step 1*	
APM fluid intelligence	0.00
*Step 2*	
BFQ Extraversion	0.28**
BFQ Agreeableness	0.18*
BFQ Consciousness	0.15
BFQ Emotional Stability	0.25**
BFQ Openness	0.22**
*Step 3*	
MSCEIT Total Score	0.32**
*R*^2^ *step 1*	0.00
*ΔR*^2^ *step 2*	0.18***
*ΔR*^2^ *step 3*	0.12***
*R*^2^ *total*	0.30***

The contributions of fluid intelligence (APM), personality traits (BFQ), and ability-based emotional intelligence (MSCEIT) to perceived social support (MSPSS). N = 149, *p < 0.05, **p < 0.01, ***p < 0.001.

Fluid intelligence did not account for significant variance at the first step. At the second step, personality traits accounted for 18% of the variance; at the third step, ability based EI accounted for an additional 12%. The overall model explained 30% of variance.

In the regression model the following personality traits are significant: Extraversion (β = 0.28, *p* < 0.01), Emotional Stability (β = 0.25, *p* < 0.01), Openness (β = 0.22, *p* < 0.01), Agreeableness (β = 0.18, *p* < 0.05). The MSCEIT Total Score is also significant (β = 0.32, *p* < 0.01).

## Discussion

The aim of the present study was to analyze the role of personality traits and ability based EI after controlling for the effects of fluid intelligence in relation to perceived social support in Italian high school students. Given the interest in promoting EI as a preventive resource (Hage et al., 2007; Kenny and Di Fabio, 2009; Di Fabio and Kenny, 2011, 2015; Kenny et al., 2014), we were interested in verifying the contribution of EI to social support beyond the effect of more static personality traits.

The results confirmed the first hypothesis as personality traits explained a significant percentage of variance in relation to perceived social support. As in prior research (Sarason et al., 1983, 1986; Di Fabio and Kenny, 2012b), personality was related to perceived social support. More specifically, the current study suggests that more extraverted, agreeable and emotionally stable people perceive a greater level of available social support.

The second hypothesis was also confirmed as ability based EI added significant incremental variance beyond that accounted for by personality traits in relation to perceived social support. These results are also consistent with existing literature (Di Fabio and Kenny, 2012b), documenting the relationship of ability based EI with perceived social support. These findings suggest that people who possess greater ability in perceiving, understanding, and managing emotions and in using emotions to facilitate thought also perceive more available social support (Di Fabio and Kenny, 2012b).

Despite the contributions of the present study in affirming existing research on the relationship of ability based EI with perceived social support, it is necessary to highlight some limitations. A first limitation is the exclusive use of a group of Italian high school students (ages of 18–20) attending the last year of school from the region of Tuscany, who are not representative of the overall Italian high school context. Future research should consider a group of participants who are more representative of the Italian population, including high school students from other geographical areas in Italy. Furthermore, future research could also involve other groups such as university students and workers. The results of the present study could also be replicated in other international contexts. Another limitation is relative to the fact that the present research is a cross-sectional study. In future research, longitudinal studies could be useful to assess the directionality of effects.

Notwithstanding the above-mentioned limitations, the results of the present study add to existing reaserch by providing an in-depth look within an Italian context at the role of ability based EI in perceived social support, beyond the effects of cognitive ability and personality. If the results of the present study are further confirmed in future research, this could also suggest the need for more intervention that seeks to build EI through specific training (Di Fabio and Kenny, 2011) and thus foster positive development and enhanced resiliency (Hage et al., 2007; Di Fabio and Kenny, 2011, 2015; Kenny et al., 2014).

Such training would be aligned with the growing interest in developing effective relational skill-building interventions for promoting positive development and functioning (Blustein and Di Fabio, 2013). Consistent with the PLS&RM framework and a prevention and positive development agenda (Hage et al., 2007; Di Fabio, 2014a; Kenny et al., 2014; Di Fabio and Kenny, 2015), group-level and systemic programs could serve to prevent relational conflict and enhance relational support (Blustein and Di Fabio, 2013). Furthermore, training specifically developed to increase EI represents an example of a relational skill-building approach that is evidenced-based.
